# Wide Resection, Extracorporeal Radiotherapy, Ipsilateral Vascularized Fibula Transposition, and Internal Fixation in a Case of Tibia Diaphyseal Ewing's Sarcoma

**DOI:** 10.7759/cureus.33526

**Published:** 2023-01-09

**Authors:** Recep Ozturk, Muzaffer Altundağ

**Affiliations:** 1 Orthopedics, Dr. Abdurrahman Yurtaslan Ankara Oncology Training and Research Hospital, Ankara, TUR; 2 Radiation Oncology, Dr. Abdurrahman Yurtaslan Ankara Oncology Training and Research Hospital, Ankara, TUR

**Keywords:** novel technique, ipsilateral graft, free vascularized graft, extracorporeal radiotherapy, ewing’s sarcoma

## Abstract

A 24-year-old female patient was referred to our hospital with the diagnosis of Ewing's sarcoma localized in the left distal tibia. Neoadjuvant chemotherapy was completed for the patient who had localized disease. En-bloc resection of the tumor segment in the diaphyseal tibia, intraoperative extracorporeal radiotherapy, and then re-implantation of the segment after clearing the tumor were performed. Transfer of the ipsilateral pedicled fibula to the medulla of the irradiated segment was performed. As far as we know, the simultaneous application of extracorporeal radiotherapy and the re-implantation method after resection of the tibial tumoral segment and the transfer of the ipsilateral fibula with its pedicle has not been previously reported in the literature. In this case, this new technique was accompanied by a satisfactory result.

## Introduction

Reconstruction is an important surgical challenge in the presence of segmental tibial loss due to tumor, trauma, or infection. In addition, the treatment process can bring a great burden to the patient physically, psycho-socially, and economically. Major tibial defects require modern reconstruction techniques and sometimes even soft tissue covering procedures [1].

In the presence of a tibial segmental defect, providing a viable bone to bridge skeletal loss is a good option to preserve limb length and function but poses several challenges. Although vascularized fibula transfer from the opposite leg is one of the options, it may cause ankle pain, instability, peroneal nerve damage, and progressive valgus deformity at the donor site [2]. In contrast, pedicled transfer of the ipsilateral fibula to the tibial defect has all the advantages of a vascularized fibula graft without these risks [1,2].

The re-implantation technique after intraoperative extracorporeal irradiation of the tumorous bone can be performed alone or by placing a vascularized fibula in its center [3]. In this study, en-bloc resection of the tumor segment in the diaphyseal tibia, intraoperative extracorporeal radiotherapy, and subsequent re-implantation of the segment after removal of the tumor were described. In addition, the transfer of the ipsilateral pedicled fibula to the medulla of the irradiated segment was performed. To the best of our knowledge, the combined application of extracorporeal radiotherapy and ipsilateral fibula transfer procedures in malignant tumors located in the tibial diaphysis is the first in the literature.

## Case presentation

**Figure 1:**
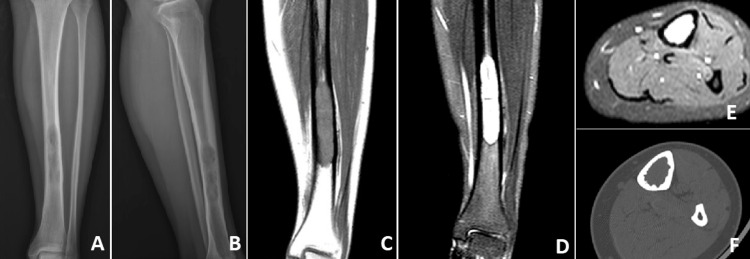
Preoperative images a) anterior-posterior radiograph, b) lateral direct radiograph, c) MRI T1 coronal section, d) MRI T2 coronal section, e) MRI T2 axial section, and f) axial tomography section MRI: Magnetic resonance imaging

A 24-year-old female patient was referred to our hospital with the diagnosis of Ewing's sarcoma localized in the left distal tibia. Neoadjuvant chemotherapy (CT) was completed in the patient who had localized disease at the time of diagnosis, and radiological images of the patient after CT showed an intramedullary tumoral lesion located distal to the left tibia diaphysis (Figure 1).

No other foci were found in staging studies. After the preparations for anesthesia were completed, the patient was placed in the supine position, and a longitudinal incision of approximately 20 cm was made extending along the lateral left cruris. The fibula was reached between the soleus and peroneus longus muscles. The flexor hallucis muscle was found and preserved. The fibular artery and vein were found. Osteotomy was performed on the proximal and distal part of the fibula, the vessels were ligated at the distal osteotomy line, the fibula was lifted together with the artery-vein package, and the fibular artery and vein were dissected up to their proximal origins. After retraction of the fibular bone, the exposed tibia was reached. The tumoral lesion was resected en bloc. Then, the resected tibia was divided into two longitudinal parts and the medulla was completely curetted and the tumoral tissues were removed.

Then, the cleaned tibia was sent to extracorporeal radiotherapy. For radiotherapy, the tissue segment was covered with a one cm bolus (tissue equivalent material). A linear accelerator device and 6 MV photon energy were used. It was irradiated with a dose of 50 Gy for approximately 30 minutes by giving midline depth from the anterior-posterior areas.

**Figure 2:**
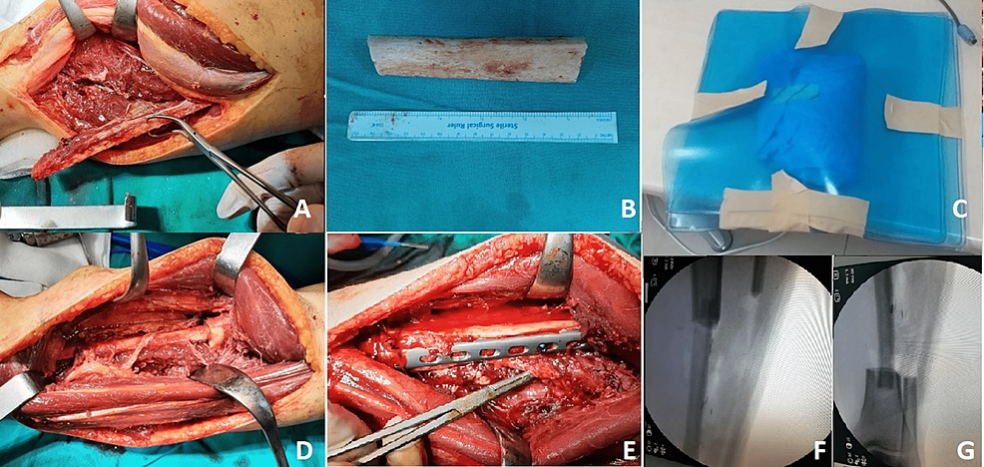
Intraoperative images a) dissection and retraction of the vascularized fibula, b) tibial tumoral segment osteotomized, c) tibial segment packed for radiotherapy, d) ipsilateral fibula placed in the defect with its pedicle, e) irradiated tibia was placed in its anatomical location and stabilized with a plate, f) scope image of the ipsilateral pedicled fibula placed in the proximal tibial medulla, and g) scope image of the ipsilateral pedicled fibula placed in the distal tibial medulla

The existing proximal and distally liberated fibula segment was placed into the defect in the tibia without damaging the feeder-artery-vein package, and the ends of the fibula segment were placed in the tibial medulla proximal and distally. Then, the irradiated tibia segment, which was cleared of tumoral tissues, was placed in its anatomical location and surrounded the vascularized fibula segment. A small hole was made on the tibial segment to prevent compression on the vessels of the fibula graft. Then, one distal tibial plate was placed and stabilized with screws to the proximal and distal tibia. In addition, the irradiated tibia was stabilized to the plate with two screws (Figure 2).

**Figure 3:**
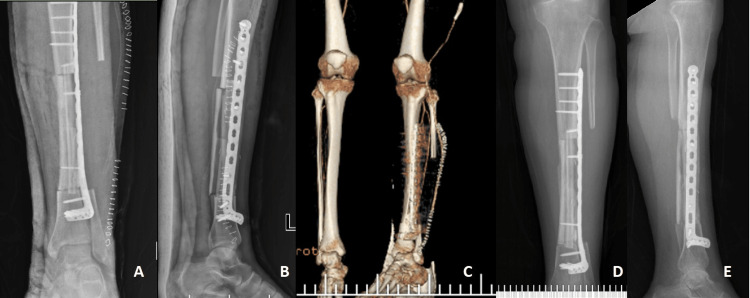
Postoperative images a) anterior-posterior radiograph, b) lateral direct radiograph, c) CT angiography found that the fibular artery was working, d) 12-month anterior-posterior radiograph, and e) 12-month lateral direct radiograph

Then, a drain was placed in the operation lodge, the layers were closed in the anatomical plane, and the extremity was placed in a short leg splint. The patient was mobilized without any weight on the first postoperative day. The patient's splint was removed in the third week. In the CT-angiography images taken in the third week, it was seen that the left fibular artery and vein, which were transferred to the tibia medulla together with the fibula, were working. The patient received adjuvant CT. Partial weight-bearing was allowed at three months postoperatively. Gradually, the load on the left tibia was increased and the patient was followed up to give full weight. The patient started full weight bearing without support in the ninth month. No recurrence or metastasis was detected in the 12-month follow-up, and the patient is satisfied with her life (Figure 3).

## Discussion

Although there are many reconstruction options for segmental tibial defects, each has its advantages and disadvantages. The use of allografts is beneficial in areas with good blood supply and small defects, but it takes a long time for union and load bearing [1]. It also has high failure rates for reasons such as fracture, nonunion, and infection. The lengthening technique with bone transport using the Ilizarov apparatus is often insufficient for adequate lengthening and is associated with important problems such as pin site infection [1,4-6]. Another technique that has become increasingly popular in massive intercalary defects in recent years is the Masquelet technique. Biau et al. used this technique in a case of Ewing's sarcoma with a 16 cm defect and reported a successful outcome [7]. They stated that this technique can also be used in the reconstruction of massive intercalary defects. However, the disadvantage of this technique is that it requires two invasive surgeries. This may delay adjuvant treatments in tumors such as osteosarcoma and Ewing's sarcoma.

Intraoperative irradiation and re-implantation of the tibial tumoral segment after resection provide a stable reconstruction that fits perfectly into the defect. It does not have problems such as breakage of the allograft, supply problems, expensiveness, or rejection of the allograft, which are experienced in allografts [8]. In addition, recurrence rates after the intraoperative extracorporeal radiotherapy technique are similar to all other extremity-sparing techniques. The area of ​​common concern in this re-implantation technique and allograft technique is avascular necrosis or resorption of the segment [3]. Therefore, it is recommended to apply a vascularized fibula to support the reconstruction [3,8].

The use of allografts in tibial segmental tumoral defects or the reconstruction of the tumor bone by intraoperative removal of the tumor, irradiation, or inactivation with agents such as alcohol has been reported [9]. In addition, placement of a living bone in the medulla is possible by vascularized transport of the ipsilateral or contralateral fibula. Li et al. used a contralateral vascularized fibula graft in three cases and an ipsilateral vascularized fibula graft in five cases of eight tibial malignancies in which they used massive allografts [10]. One of the two advantages of the group in which they used ipsilateral pedicled fibula graft was the absence of donor site complications and the second was the shorter surgical time. In this study, en-bloc resection of the tumor segment in the diaphyseal tibia, intraoperative extracorporeal radiotherapy, and then re-implantation of the segment after clearing the tumor were performed. Transfer of the ipsilateral pedicled fibula to the medulla of the irradiated segment was performed. As far as we know, the simultaneous application of extracorporeal radiotherapy and the re-implantation method after resection of the tibial tumoral segment and the transfer of the ipsilateral fibula with its pedicle has not been previously reported in the literature. In this case, this new technique was accompanied by a satisfactory result.

## Conclusions

Vascularized bone transfer and reimplantation of the diaphyseal tumoral segment are important options in many cases where durable, long-term stable reconstruction is required. The simultaneous application of extracorporeal radiotherapy and the re-implantation method after resection of the tibial tumoral segment and the transfer of the ipsilateral fibula with its pedicle has not been previously reported in the literature, and this technique was accompanied by a satisfactory result without involving donor site and allograft complications.
